# S6K1 and 4E-BP1 Are Independent Regulated and Control Cellular Growth in Bladder Cancer

**DOI:** 10.1371/journal.pone.0027509

**Published:** 2011-11-15

**Authors:** Roman Nawroth, Florian Stellwagen, Wolfgang A. Schulz, Robert Stoehr, Arndt Hartmann, Bernd J. Krause, Juergen E. Gschwend, Margitta Retz

**Affiliations:** 1 Department of Urology, Klinikum rechts der Isar der Technischen Universität München, Munich, Germany; 2 Department of Pathology, University Hospital Erlangen, Erlangen, Germany; 3 Department of Urology, Heinrich-Heine-University, Düsseldorf, Germany; 4 Department of Nuclear Medicine, University of Rostock, Rostock, Germany; Florida International University, United States of America

## Abstract

Aberrant activation and mutation status of proteins in the phosphatidylinositol-3-kinase (PI3K)/Akt/mammalian target of rapamycin (mTOR) and the mitogen activated protein kinase (MAPK) signaling pathways have been linked to tumorigenesis in various tumors including urothelial carcinoma (UC). However, anti-tumor therapy with small molecule inhibitors against mTOR turned out to be less successful than expected. We characterized the molecular mechanism of this pathway in urothelial carcinoma by interfering with different molecular components using small chemical inhibitors and siRNA technology and analyzed effects on the molecular activation status, cell growth, proliferation and apoptosis. In a majority of tested cell lines constitutive activation of the PI3K was observed. Manipulation of mTOR or Akt expression or activity only regulated phosphorylation of S6K1 but not 4E-BP1. Instead, we provide evidence for an alternative mTOR independent but PI3K dependent regulation of 4E-BP1. Only the simultaneous inhibition of both S6K1 and 4E-BP1 suppressed cell growth efficiently. Crosstalk between PI3K and the MAPK signaling pathway is mediated via PI3K and indirect by S6K1 activity. Inhibition of MEK1/2 results in activation of Akt but not mTOR/S6K1 or 4E-BP1. Our data suggest that 4E-BP1 is a potential new target molecule and stratification marker for anti cancer therapy in UC and support the consideration of a multi-targeting approach against PI3K, mTORC1/2 and MAPK.

## Introduction

Bladder cancer is the fifth most common cancer world-wide with an estimated 357.000 new cases and 145.000 deaths in 2010 [1]. Urothelial carcinoma (UC) representing 90% of all bladder tumors are a heterogenous entity comprised of papillary tumors and solid invasive carcinomas which require radical treatment once they have progressed into the muscular layer of the bladder. If untreated, about 85% of patients with invasive bladder tumor will die from disease progression within two years from diagnosis [2]. Radical cystectomy with pelvic lymph node dissection is the standard of care for muscle-invasive bladder cancer. With this approach 5-year progression-free survival probabilities of 65–68% can be achieved across all tumor stages [3], [4]. Despite advances in cisplatin-based chemotherapy for patients with metastatic disease, the median overall 5-year survival time is limited to 14–15 months [5]. Thus, new therapeutic approaches are highly desirable. Better knowledge about aberrant activation of cell signaling pathways that are involved in tumorigenesis of the bladder might provide suitable molecular targets for novel therapies [6], [7], [8]. One such pathway is the PI3K/Akt/mTOR signaling pathway that has been linked to tumorigenesis in many tissues [9], [10].

Normally, upon activation by tyrosine receptor kinases or RAS proteins PI3K converts phosphatidylinositol-4,5-bisphosphate (PIP_2_) into phosphatidylinsositol-3,4,5-trisphosphate (PIP_3_). This reaction can be reversed by the PI3K antagonist PTEN (phosphatase and tensin homologue deleted on chromosome 10). PIP_3_ recruits PDK1 (protein dependent kinase 1) to the cell membrane where it binds and phosphorylates Akt at amino acid residue T308. Akt is considered as the most critical signaling node in this pathway regulating various substrates affecting cellular processes involved in the control of cell growth and survival [11]. Akt signaling converges through the tuberous sclerosis proteins 1/2 (TSC1/2) and the small GTPase Rheb on mTOR, a serine/threonine kinase that forms two distinct protein complexes with either raptor (regulatory-associated protein of mTOR) yielding mTORC1 or rictor (rapamycin insensitive companion of mTOR) yielding mTORC2 [10]. mTORC2 can be activated by PI3K directly and phosphorylates Akt at S473, which together with phosphorylation at T308 results in the full activation of Akt [12], [13]. Akt phosphorylated at S473 has been associated with poor prognosis in many cancers including those of the pancreas, liver, prostate and breast [13]. The best-defined substrates of mTORC1 are the kinases p70S6K1 (S6K1) and eIF4E-binding protein 1 (4E-BP1), both of which are important in the control of protein translation initiation [14], [15], [16]. Dephosphorylated 4E-BP1 can bind to the elF4E protein complex to prevent cap-dependent translation and plays an important role in mediating signaling events of the PI3K and MAPK pathway in tumors [15]. Phosphorylated S6K1 is required for translation of 5′ terminal oligopyrimidine (TOP) mRNAs. Dephosphorylation of S6K1 results in a feedback loop that results in upregulation of receptor tyrosine kinases or insulin receptor substrate proteins (IRS), which then activate the PI3K and also the MAPK signaling pathway [17], [18]. Crosstalk between the PI3K and the MAPK signaling network occurs also by way of RAS and ERK1/2, activating PI3K and additionally mTORC1 through TSC1/2 [19], [20], [21], [22].

mTORC1 can be selectively inhibited by rapamycin, a macrolide antibiotic, that in a complex with the cytosolic protein FKBP12 inhibits mTORC1 and at higher concentrations also mTORC2 [23], [24]. Novel mTOR inhibitors in clinical use such as everolimus (RAD001) or temsirolimus (CCI-779) are derivatives of rapamycin. Administered as anti-tumor drugs, the response rates in patients differ from 0–30% dependent on the tumor [25]. It is suspected that the major limitation in the clinical application of mTOR inhibitors results from the mTORC1-S6K1-PI3K feedback loop. This resulted in the development of inhibitors that target both, PI3K and mTOR, such as NVP-BEZ235 an imidazo[4,5-c]quinoline derivative [24].

In UC, genetic alterations in the PI3K pathway are common and occur mostly in PIK3CA, PTEN, Akt or TSC1/2 [26]. PTEN deletions and mutations as well as diminished protein expression are found especially at higher tumor stages and grades. Phosphorylated S6K1 is an independent predictor of disease-specific survival [27], [28]. In preclinical studies with UC cell lines, PI3K inhibition by LY294002 exhibited dependent on the cell line used minimal to 40% reduction on proliferative effects and blocked cell invasion [29], [30], [31]. Reconstitution of PTEN suppressed tumor growth and genetic silencing of Akt resulted in increased radiosensitivity of the tumor cells [27], [32]. The potential application of rapamycin derivatives in UC therapy was supported by their ability to reduce cell viability in various bladder cancer-derived cell lines and in a mouse model of progressive bladder cancer in which p53 and PTEN are deleted in the bladder urothelium [9], [33], [34], [35], [36]. Despite such promising preclinical observations, preliminary results from a single-arm, phase II trial with everolimus as monotherapy showed only modest antitumor activity in patients with metastatic UC [37]. Data on the functional role and molecular mechanism of the PI3K/Akt/mTOR and MAPK signaling pathways in UC are very limited to date and can not explain clinical results. Thus, we characterized the molecular mechanism of the PI3K/AKT/mTOR signaling pathway and its cross-regulation with the MAPK pathway in vitro in cell lines harbouring typical mutations for UC. Functional consequences on cell signaling events and cell growth were studied after pharmacological or genetic interference with different molecular components of this pathway via small molecule inhibitors or siRNA/shRNA oligonucleotides. Our findings provide new insights into the molecular network of the signaling pathways examined that result in a rationale for new treatment strategies and suggests candidate proteins for molecular stratification for patients suffering from metastatic UC.

## Materials and Methods

### Cell lines and reagents

The cell lines RT4, HT1376, 647V, 486P, J82, 253J, EJ28, T24 (American Type Culture Collection, Rockville, MD, USA) and RT112 (German Collection of Microorganisms and Cell Cultures) were maintained at 37°C in RPMI 1640 or DMEM supplemented with 10% FCS in a humidified atmosphere containing 5% or 7,5% CO_2_. NVP-BEZ235 and RAD001 were kindly provided by Novartis Pharma. U0126 was purchased from Cell Signaling Technology and LY294002 from Promega. Stock solutions were prepared in DMSO. Working solutions were freshly prepared in cell culture medium at depicted concentrations.

### Constructs, transfection and infection

siRNA oligonucleotides against 4E-BP1, S6K1 and control were designed by and purchased from Qiagen GmbH and Akt 1,−2,−3 stealth siRNA oligonucleotides from Invitrogen. Transient transfections were performed using X-treme gene transfection or lipofectamin reagent (Roche Diagnostics, Invitrogen) according to the manufacturer's instructions, using 2 µg/siRNA/6-well. For triple transfection adenovirus mTOR shRNA and control shRNA was purchased from SIRION Biotech. 1.5×10^5^ cells were seeded on 6-well plates one day before infection and adenovirus particles were added at a multiplicity of infection of 30. Suppression of protein expression was analyzed by immunoblots 2–6 days after transfection or infection.

### Immunoblotting and Antibodies

Cells were lysed in RIPA buffer (150 mM NaCl, 50 mM Tris-HCl pH 7.2, 1% Triton X-100, 0.1% SDS, protease inhibitor cocktail (Roche Diagnostics), phosphatase inhibitor Mix (SERVA) for 20 min on ice. Insoluble material was pelleted by centrifugation for 30 min at 4°C, 30,000 g. Protein concentration was quantified with the BCA™ Protein Assay (Pierce). Equal amounts of protein were subjected to SDS-PAGE on 7.5–12% polyacrylamide gels and transferred to PVDF membrane (Zefa). For immunoblotting, the antibodies used included: Akt (C67E7), Akt1, Akt2, Akt3, Phospho-Akt (Ser473), Phospho-Akt (Thr308), GSK3β, Phospho-GSK3β (Ser9), mTOR, Phospho-mTOR (Ser2448), 4E-BP1, Phospho-4E-BP1 (Thr37/46), S6K1, Phospho-S6K1(Thr389), S6RP, Phospho-S6RP, p44/42 MAPK, Phospho-p44/42 MAPK, c-Raf, Phospho-c-Raf (Ser338) (Cell Signaling Technologies). Secondary HRPO conjugated antibodies were purchased from Dianova. Immunoblotting was performed as described previously or modified according to the manufacturer's recommendation [38]. ECL-reaction was visualized using Hyperfilm ECL autoradiography films (GE Healthcare). For quantification, the chemiluminescence signal was analyzed via the ChemiDoc™ XRS and Quantity One® Software from Bio-Rad Laboratories, Inc.

### Cell growth

To evaluate cell growth, cells were seeded at 1000 cells/96-well and treated as described. After harvesting, cells were stained with trypane blue and living cells were counted in a Neubauer chamber.

### Cell proliferation

Cell proliferation was measured 24 h after treatment of cells with RAD001, NVP-BEZ235 and U0126 or 48–72 h after transfection/infection with siRNAs/shRNAs. Bromodeoxyuridine (BrdU) incorporation for detection of newly synthesized DNA and 7-amino-actinomycin D (7-AAD) staining for detection of double stranded DNA was performed using an APC BrdU Flow Kit (BD Biosciences) by pulsing cells for two hours according to the manufacturer's protocol followed by flow cytometry analysis.

### Apoptosis assay

Apoptosis was detected by measuring activity of caspase 3/7 using the Caspase-Glo 3/7 Assay from Promega GmbH, according to the manufacturer's protocol.

### Cell viability

Cells were treated with RAD001, NVP-BEZ235 and U0126 in 96-well plates. At 36 h, cells were incubated for 3 h with XTT. The enzymatic reaction was analyzed at 450 and 650 nm in an ELISA reader.

### Statistical analysis

Student T-test was used to compare the means in different groups. Statistical calculations were done using Microsoft Office Excel.

## Results

### Expression and activation status of the PI3K/Akt/mTOR pathway in urothelial carcinoma cell lines

**Figure 1:**
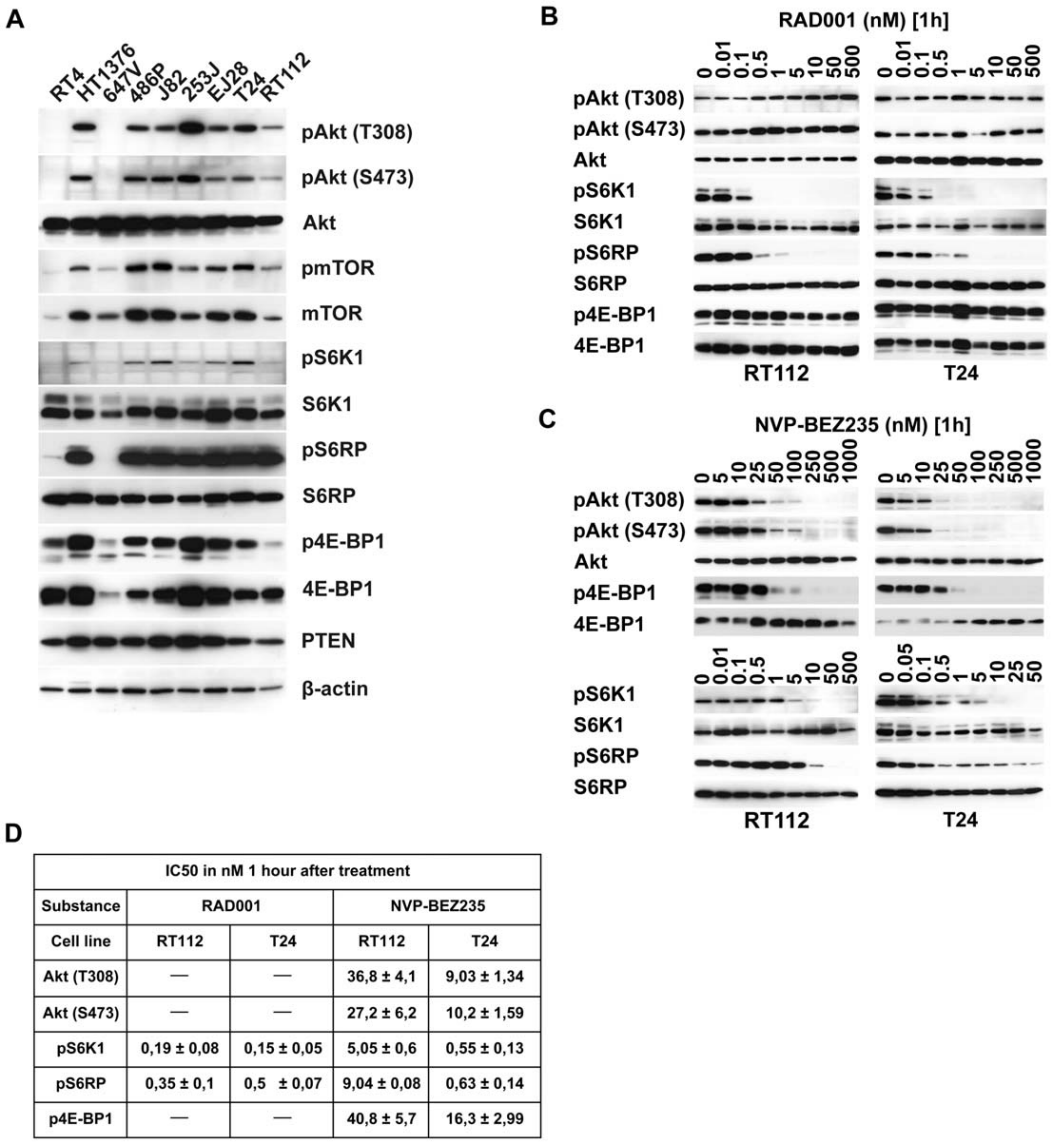
Activation status of the PI3K signaling pathway and cellular profiling of RAD001 and NVP-BEZ235. **A:** For protein analysis, cells were lysed in RIPA buffer applied to SDS-PAGE and transferred to PVDF membrane. Phosphorylation and expression status of proteins were analyzed in immunoblots. **B, C:** Cell lines were treated for 1 h with the indicated concentrations of RAD001 and NVP-BEZ235. The control (0) contained same concentrations of DMSO as in samples with chemical compounds. One representative result from 3 independent experiments is shown. **D:** The inhibitory concentration of 50% (IC50) was determined for target proteins of PI3K and mTORC1. Chemiluminescence signals were quantified using the ChemiDoc imaging system (BioRad Laboratories) and normalized to protein expression level. The average values from three or more independent experiments are shown.

We analyzed expression and activation status of molecular key components in the PI3K pathway in a panel of 9 UC derived cell lines. Cells were harvested and lyzed for protein analysis in a subconfluent stage. Expression of Akt, mTOR, S6K1, S6RP, 4E-BP1 and PTEN was detected in all cell lines (Figure 1A). Phosphorylation of Akt at T308 and S473 and mTOR, 4E-BP1, S6K1 and S6RP was observed in 7 out of 9 cell lines. No activation of Akt could be detected in RT4 and 647V cells. In these two cell lines mTOR, S6K1 and S6RP phosphorylation was significantly reduced, whereas 4E-BP1 was still phosphorylated in RT4 cells. Thus, the majority of the examined cell lines exhibited a constitutively activated PI3K/Akt/mTOR signaling pathway. Expression of PTEN does not prevent this activation under the growing conditions examined. For further experiments we decided to work with the two UC cell lines RT112 (overexpressing FGFR3) and T24 (oncogenic H-Ras, homozygous PTEN mutation), both exhibiting frequently found genomic alterations in bladder cancer [39], [40], [41].

### RAD001/CCI-779 and NVP-BEZ235 block PI3K and mTOR downstream targets in a dose-dependent manner – mTORC1 independent regulation of 4E-BP1

In order to characterize the functional relevance of this pathway in UC we used the rapamycin derivatives RAD001 and CCI-779 and the PI3K-inhibitor NVP-BEZ235. Since effects of CCI-779 and RAD001 treatment were identical we only depict RAD001 results. First, dose response assays were conducted to assess the activity of RAD001 and NVP-BEZ235 1 h after cellular treatment. Activity of the compounds was characterized by the phosphorylation pattern of S6K1/S6RP/4E-BP1 for both substances and in addition for Akt when using NVP-BEZ235. Signal intensities were measured by a chemiluminescence imaging system and inhibitory concentrations of 50% (IC50) for phosphorylation level normalized to the protein level were calculated (Figure 1B,D). RAD001 inhibited phosphorylation of S6K1 and S6RP but not of 4E-BP1 (Figure 1B). When exposing cells to NVP-BEZ235, inhibition of S6K1/S6RP was observed at lower IC50 concentrations than those necessary for inhibiting Akt phosphorylation at T308/S473 (Figure 1C,D). 4E-BP1 phosphorylation was greatly diminished at concentrations that correlated with those necessary for Akt inhibition. Thus, inhibition of mTORC1 by rapamycin derivatives regulated only S6K1/S6RP phosphorylation whereas inhibition of PI3K and mTORC1/2 inhibited both, S6K1 and 4E-BP1/S6RP phosphorylation. T24 cells responded at similar concentration to RAD001 treatment as RT112 but 3 times as sensitive to NVP-BEZ235 treatment.

### Long-term treatment with RAD001 and NVP-BEZ235 results in S6K1 mediated hyperactivation of PI3K/Akt

**Figure 2:**
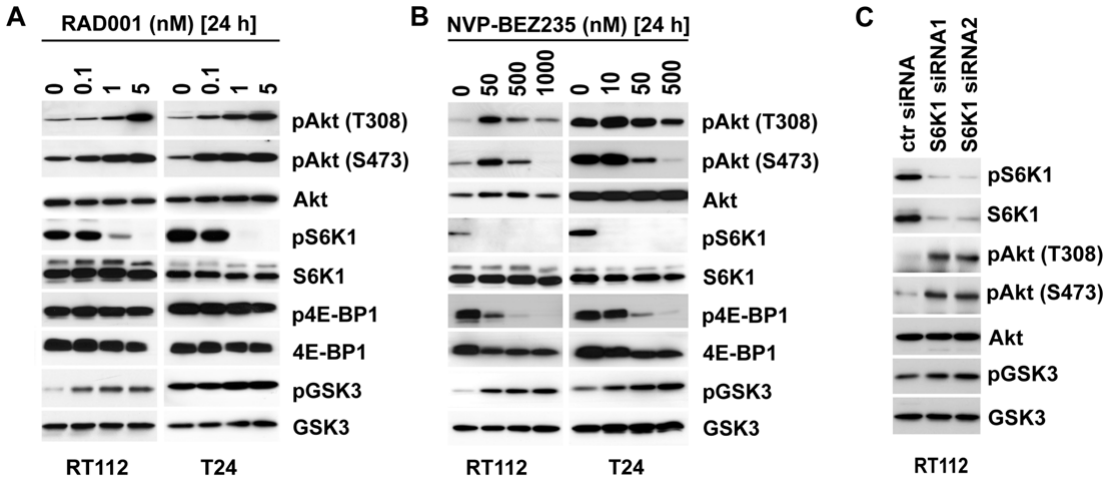
Long term treatment of cells with RAD001 and NVP-BEZ235 - S6K1 mediated activation of Akt. **A, B:** Effect of RAD001 or NVP-BEZ235 treatment over 24 hours on phosphorylation level of Akt, S6K1, 4E-BP1 and GSK3-β in RT112 and T24 cells. Whole cell lysates were applied to SDS-PAGE and blotted on PVDF membranes followed by immunoblots to detect expression and phosphorylation level of depicted proteins. The control (0) contained same concentration of DMSO as in samples with chemical compounds. **C:** RT112 cells were transfected with two independent S6K1 specific siRNA oligonucleotides and one random siRNA oligonucleotide as control (ctr siRNA). Three days after transfection, expression and phosphorylation level of S6K1, Akt and GSK3-β were analyzed in immunoblots.

It has been described that S6K1 activation results in a negative feedback loop regulating not only PI3K activity which is probably responsible for the limited success of rapamycin derivatives in clinical use [17]. Thus, we addressed the effect of the compounds used on this feedback loop. We used three different concentrations of RAD001 and NVP-BEZ235 that resulted in approximately 50% to 100% inhibition of their molecular targets and analyzed effects on PI3K signaling pathway activation over a period of 72 hours. RAD001 treatment resulted in S6K1 dephosphorylation and induced hyperphosphorylation of Akt on T308 and S473 in a concentration dependent manner throughout the observation period (1–72 h) in RT112 cells whereas in T24 cells Akt hyperactivation was induced only 24–72 h after treatment (Figure 1B/C, 2A, data for 72 h not shown). The activation status of the Akt downstream target GSK3-β correlated with the phosphorylation level of Akt in both cell lines (Figure 2A). 4E-BP1 phosphorylation or protein level was not affected by RAD001 treatment. When using NVP-BEZ235 the initial dephosphorylation of Akt 1 h after treatment reversed after 24 h and even concentrations 100-fold above the IC50 were not sufficient to prevent phosphorylation on T308 and only partially on S473 (Figure 2B). Corresponding to the activation status of Akt, phosphorylation of the Akt substrate GSK3-β was observed. Notably, repeated treatment with freshly prepared NVP-BEZ235 1 h before harvesting the cells did not prevent this hyperactivation of Akt (data not shown). Phosphorylation of 4E-BP1 remained suppressed throughout the observation period (Figure 2C).

Whether S6K1 directly induced PI3K/Akt activity was addressed by silencing S6K1 expression using two specific siRNAs directed against S6K1. Two days after transfection, western blot analyses demonstrated a 90–96% reduction in S6K1 protein level (Figure 2C). The downregulation of S6K1 expression correlated with hyperphosphorylation of Akt at S473 and T308 and GSK3-β, supporting the described S6K1-PI3K/Akt feedback loop.

### Inhibition of PI3K/mTOR but neither silencing of mTOR nor Akt expression regulates 4E-BP1 phosphorylation

**Figure 3:**
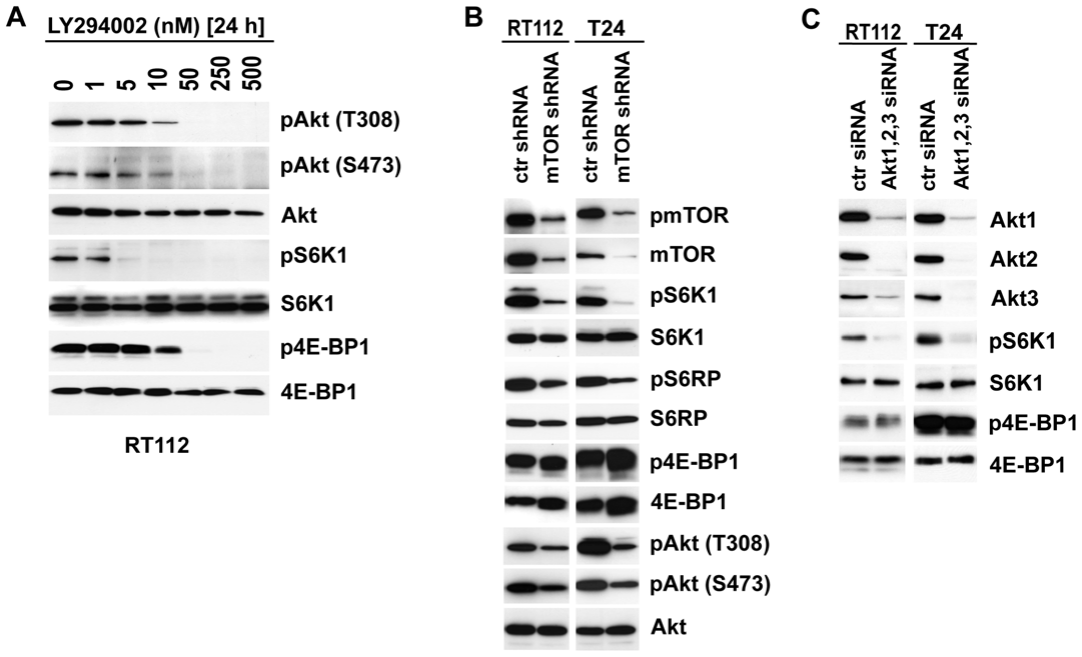
4E-BP1 phosphorylation is not regulated by Akt or mTOR expression but PI3K activity. **A:** Dose response assay with cells treated 24 hours with the PI3K inhibitor LY294002. Cell lysates were analyzed in immunoblots showing dose dependent effects on expression and phosphorylation level of Akt, S6K1 and 4E-BP1 in RT112 cells. **B:** Silencing of mTOR expression 3 days after infection with adenoviral mTOR shRNA or control shRNA (ctr shRNA). Whole cell lysates were analyzed in immunoblots for phosphorylation and expression level of S6K1, S6RP, Akt and 4E-BP1. **C:** Akt expression regulates phosphorylation of S6K1 but not 4E-BP1. Transfecting cells with specific stealth siRNA oligonucleotides or control siRNA silenced expression of all three isoforms of Akt. 3 days after transfection cells were lyzed applied to SDS-PAGE blotted to PVDF membrane and expression level of Akt isoforms and expression and phosphorylation level of 4E-BP1 and S6K1 were analyzed.

These results prompted us to characterize the signalling circuitry of PI3K, mTOR, Akt and 4E-BP1 in greater detail. In most suggested models, S6K1 and 4E-BP1 are downstream targets of Akt and mTORC1 and phosphorylation in most reports is simultaneously regulated [14], [15]. Our results demonstrate that only UC cells treated with NVP-BEZ235 but not RAD001 exhibited suppression of both S6K1 and 4E-BP1 phosphorylation suggesting that 4E-BP1 is regulated differently than S6K1. Since NVP-BEZ235 targets members of the PI3K protein family, PI3K or its downstream target Akt or mTOR might be involved in regulating 4E-BP1 phosphorylation. We first tested if we could verify the effects of NVP-BEZ235 by using the thoroughly characterized kinase inhibitor LY293002 which was originally designed as a specific PI3K inhibitor but displays also activity towards other PI3K non-related kinases [42], [43]. S6K1 phosphorylation was reduced with an IC50 of 3 nM whereas the IC50 doubled to 7 nM to induce a reduction in Akt (S473/T308) and 4E-BP1 phosphorylation, thus resembling the kinetics of NVP-BEZ235 activity on Akt/4E-BP1 phosphorylation (Figure 3A and 1B).

In order to further examine this observation, we used an adenoviral shRNA approach to silence mTOR protein expression which should result in specific inactivation of both, mTORC1 and mTORC2 protein complexes. Two days after infection, western blot analyses confirmed an 85–96% knockdown in mTOR protein level that remained stable for 5 days (Figure 3B). The knockdown resulted in dephosphorylation of S6K1 and S6RP without affecting protein expression level. Only minor effects on 4E-BP1 phosphorylation relative to 4E-BP1 protein level was detected. Notably, Akt phosphorylation was reduced by 40–60% on T308 and S473 residues.

Last, the influence of Akt on 4E-BP1 phosphorylation was characterized by using siRNAs directed against the three different isoforms of Akt. Three days after transfection, the combined knock down of all three isoforms resulted in >90% reduced protein expression of all three Akt isoforms (Figure 3C). Only S6K1 phosphorylation but not 4E-BP1 phosphorylation level were reduced without affecting protein level indicating that 4E-BP1 unlike S6K1 is not downstream from Akt in the examined cell lines.

### Crosstalk between the PI3K and MAPK signaling pathway occurs at multiple steps

**Figure 4:**
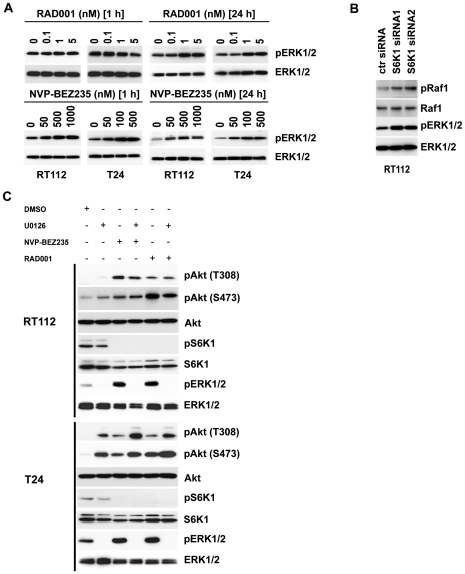
Crosstalk between the PI3K and MAPK signaling pathways. **A:** RT112 and T24 cells were treated with RAD001 or NVP-BEZ235 for 1 h or 24 h. Expression and phosphorylation status of ERK1/2 was analyzed in immunoblots on whole cell lysates. **B:** Two days after transfection with siRNA oligonucleotides against S6K1 or control siRNA (ctr siRNA), cells were lysed and phosphorylation and expression status of Raf and ERK1/2 was analyzed in immunoblots. **C:** Cells were treated for 24 h with RAD001, NVP-BEZ235 and U0126 alone or in combination and effects on expression and phosphorylation level of Akt, S6K1 and ERK1/2 was analyzed in immunoblots.

Given the importance of PI3K and MAPK pathway biology, we examined the interaction of the two pathways in bladder cancer. Cells were treated with RAD001, NVP-BEZ235 or with the MEK1/2 inhibitor U0126 and activation status of key proteins in both pathways 1–72 h after treatment were analyzed in western blots. Inhibition of mTORC1 by RAD001 induced activation of ERK1/2 phosphorylation 24–72 h after treatment (Figure 4A, upper panel). The same effect was observed when silencing S6K1 protein expression which resulted in increased Raf1/ERK1/2 phosphorylation (Figure 4B). However, inhibition of both PI3K and mTOR by NVP-BEZ235 rapidly induced ERK1/2 phosphorylation throughout the observed period of 1–72 h (Figure 4A, lower panel). We conclude that suppression of PI3K/Akt/mTOR activity generally results in activation of Raf/MEK/ERK signaling pathway, but the kinetics of the process depends on which molecular target in the former pathway is inhibited.

We then analyzed the biochemical consequences when inhibiting both the PI3K/AKT/mTOR and the MAPK pathway, using RAD001, NVP-BEZ235 or U0126 in combination or U0126 alone. When cells were treated with U0126 alone, ERK1/2 phosphorylation was completely abolished whereas Akt phosphorylation on S473 and T308 was upregulated (Figure 4C). Interestingly, we could not demonstrate any changes in S6K1 phosphorylation. When combining U0126 with either RAD001 or NVP-BEZ235, Akt hyperphosphorylation was significantly increased compared to treatment with either single substance in T24 cells.

### PI3K/AKT/mTOR and MAPK signaling regulate cell proliferation, viability and apoptosis

**Figure 5:**
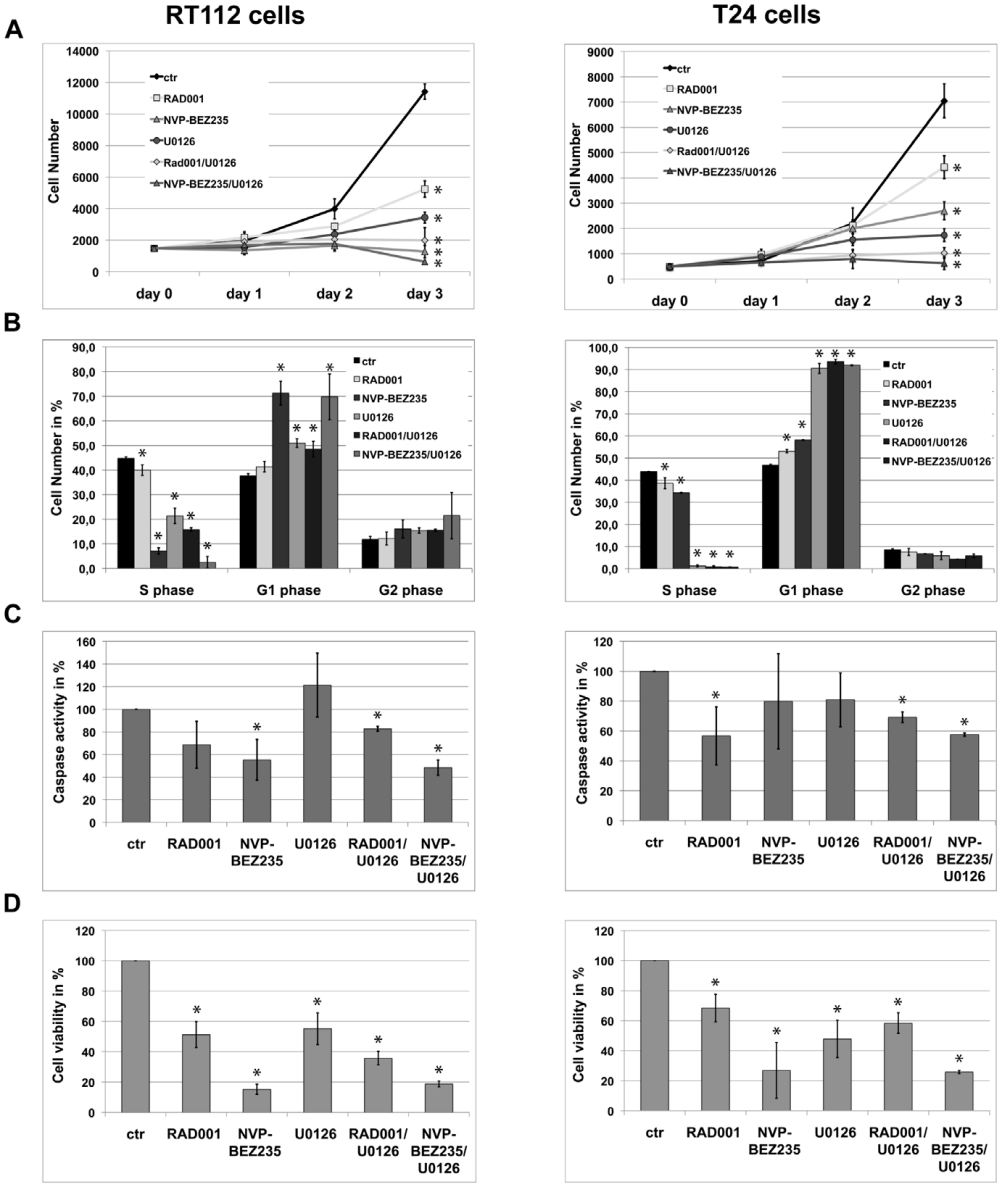
Effects of PI3K/Akt/mTOR and MAPK pathway inhibition on cell growth, proliferation, apoptosis and cell viability. RT112 and T24 cells were treated with RAD001 (5 nM), NVP-BEZ235 (100 nM for T24, 500 nM for RT112) and U0126 (25 nM) alone or the indicated combination and a DMSO control (ctr). **A:** For cell counts, cells were stained with trypan blue and numbers of living cells were determined. **B:** Cell cycle analysis after 24 h treatment with chemical compounds by BrdU incorporation in combination with 7-AAD staining. **C:** Measurement of caspase 3/7 activity as a parameter for apoptosis and **D:** XTT-test for detection of cell viability performed 24 h after treatment. Values shown are the mean ± standard deviation from 3 independent experiments. Student t-test was performed for statistic analysis (*: p<0,05).

Next we determined how manipulation of PI3K and MAPK signaling influences cellular proliferation in UC. First, cells were treated with RAD001, NVP-BEZ235 and U0126 alone or in combination and cell proliferation was monitored over time. Three days after treatment, RAD001 inhibited cell proliferation by 62% in RT112 and 40% in T24 cells relative to the solvent control (Figure 5A). Treatment with NVP-BEZ235 completely inhibited cell proliferation in RT112 cells and reduced it by 66% in T24 cells. U0126 treatment inhibited proliferation by 70% and 76% in RT112 or T24 cells, respectively. Combining RAD001 or NVP-BEZ235 with U0126 yielded additive effects, with the combination of NVP-BEZ235/U0126 being most effective (Figure 5A). In order to characterize these effects in more detail, cell cycle analysis was measured by combining incorporation of BrdU and 7-AAD staining, the apoptotic response by measuring caspase activity and viability/metabolism by XTT-assays. As for cell cycle analysis, RAD001 treatment diminished the fraction of cells undergoing S-phase by 11% in both cell lines (Figure 5B). NVP-BEZ235 reduced S-phase cells by 84% in RT112 and by 22% in T24, whereas U0126 reduced cells in S-phase by 97% in T24 but only by 52% in RT112 cells. The combination of PI3K and MAPK signaling inhibitors had additive effects on cell proliferation with the combination of NVP-BEZ235/U0126 being most effective in reducing cell numbers in S-phase by 94–98%. All substances induced an increase in cells arrested in G1 phase that correlated to the decrease in cells entering S-phase, indicating that the inhibition of either pathway results in G1 arrest in UC cell lines (Figure 5B). Apoptosis was measured by caspase activity (Figure 5C) [11]. Both, RAD001 and NVP-BEZ235 treatment decreased caspase activation in RT112 cells by 32–45%. In T24 cells, RAD001 and NVP-BEZ235 reduced caspase activity by 43–48%. U0126 had no significant effect on caspase activity and did not increase activity of RAD001 or NVP-BEZ235 when combined together. For measuring cell viability, same numbers of cells were analyzed using a XTT assay. Dependent on the cellular background, we observed a reduction of 30–50% with RAD001 and 70–85% with NVP-BEZ235 (Figure 5D). Interestingly, we could not detect additive effects by the combined inhibition of the PI3K/Akt/mTOR and MAPK pathways. We conclude that PI3K/mTOR and MAPK pathway activity in UC control cell proliferation, apoptosis and cell vitality and that both pathways can partially substitute for loss of each other with respect to proliferation.

### The combined activity of S6K1 and 4E-BP1 regulate cell proliferation in urothelial carcinoma

**Figure 6:**
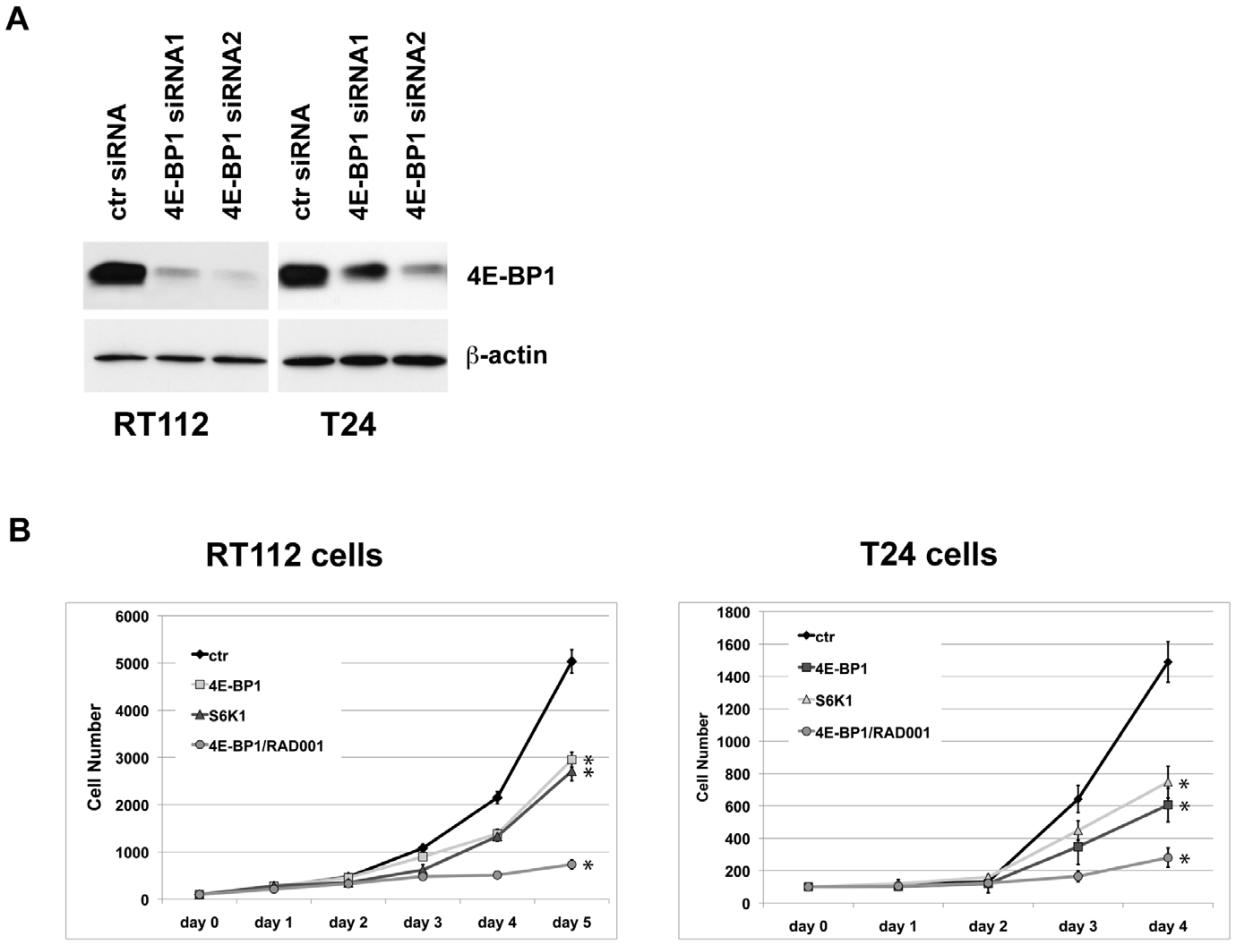
Combined activities of S6K1 and 4E-BP1 regulate cell growth. **A:** Two days after transfection with siRNAs against 4E-BP-1, protein expression in RT112 and T24 cells was detected in immunoblots. β-actin was used as a loading control. **B:** Growth of living cells, silenced for S6K1 or 4E-BP1 expression or cells silenced for 4E-BP1 expression and incubated with everolimus (RAD001) were monitored over time. Mean values ± standard deviations are shown; statistical comparisons were performed using the student t-test (*: p<0,05).

Finally, we addressed the question why NVP-BEZ235 affects cell growth more efficaciously than RAD001. We demonstrated that NVP-BEZ235 unlike RAD001 influences both, S6K1 and 4E-BP1 phosphorylation. Thus, we used either siRNA oligonucleotides directed against S6K1 or 4E-BP1 or combined RAD001 with 4E-BP1 siRNAs to mimic the presumed additional effect of NVP-BEZ235. Two days after transfection siRNAs against 4E-BP1 or S6K1 reduced protein expression by 80–96% (Figure 6A, 2C). Cells silenced for S6K1 or 4E-BP1 expression exhibited significantly reduced cell proliferation by 40% (RT112) to 60% (T24) compared to controls (Fig. 6B), yielding comparable effects to those observed after everolimus treatment (Figure 5A). However, the combination of 4E-BP1 siRNA and everolimus treatment reduced cell growth by 80–85%, the same degree as observed with NVP-BEZ235. Analysis of cell proliferation demonstrated that the effect was again mediated by cell cycle arrest in the G1/G0 phase (data not shown). In summary, both the mTORC1 downstream target S6K1 and 4E-BP1 are to a similar extent involved in regulating UC cell proliferation by controlling cell cycle progression from G1/0 to S phase and cell viability.

## Discussion

Despite a better understanding of bladder cancer biology within recent years, only minor improvements in the therapeutic management of this disease have been achieved during the last two decades. The PI3K/Akt/mTOR and MAPK signaling pathways are prone to mutations and aberrant activation in this tumor entity and might provide suitable targets for more effective therapies [44]. Accordingly, we characterized both signaling pathways in regard to activation status, molecular mechanism and relevance for cellular proliferation in UC. We could confirm previously described regulatory circuitries that control activation of these pathways. Our data strongly suggest a novel mechanism that controls activation of the downstream element 4E-BP1 within this signaling network. Also, we show evidence that 4E-BP1 might be a suitable new molecular target for cancer therapy.

**Figure 7:**
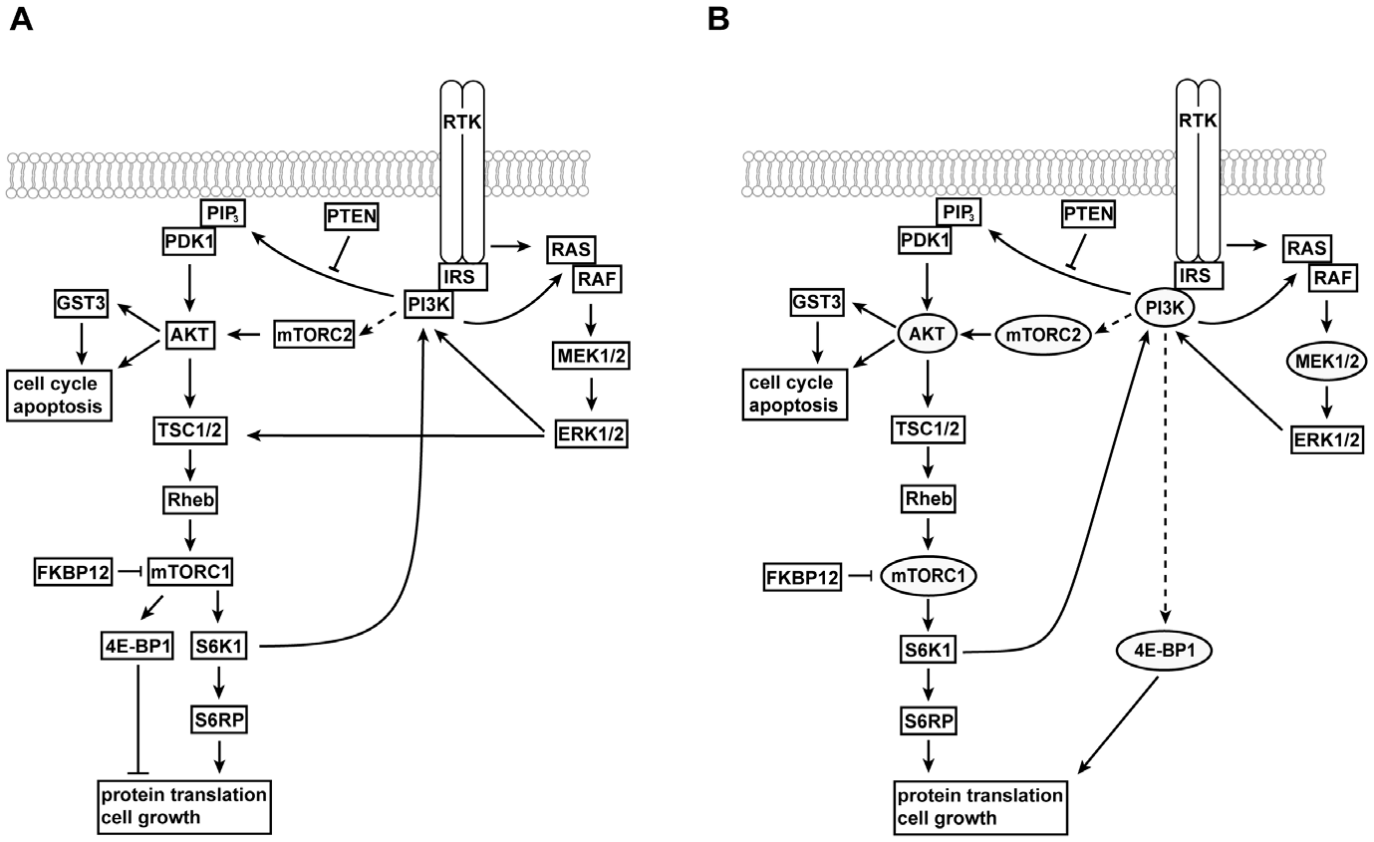
Schematic representation of the PI3K/Akt/mTOR and MAPK signaling pathways in urothelial carcinoma. **A:** In the current model, activation of PI3K results in activation of Akt via mTORC2 and PDK1, a process that can be reversed by PTEN. Subsequently, Akt regulates amongst other proteins TSC1/2 that together with RHEB control mTORC1 and its downstream targets S6K1 and 4E-BP1. Both molecules are involved in the regulation of protein translation and cell proliferation. Crosstalk between the PI3K and the MAPK pathway has been demonstrated from PI3K/IRS to RAS and from ERK1/2 to TSC1/2 and PI3K. **B:** According to the present results, in urothelial carcinoma mTORC1 regulates activity of S6K1/S6RP whereas 4E-BP1 activation status is regulated via PI3K. Both, 4E-BP1 and S6K1 are involved in regulating cell proliferation. Inactivation of PI3K and also S6K1 results in phosphorylation of RAS and ERK1/2. The inhibition of MEK1/2 induces Akt but neither S6K1 nor 4E-BP1 phosphorylation. Potential target molecules and stratification marker are shaped elliptical.

Both signaling pathways are constitutively activated in the majority of the examined UC derived cell lines supporting their importance in UC [41], [44] (Figure 1). When interfering with the PI3K pathway by using either mTORC1 (RAD001, CCI779) or PI3K/mTOR inhibitors (NVP-BEZ235) we observed that mTORC1 inhibitors were less efficient on suppressing cellular growth compared to the PI3K/mTOR inhibitor. By characterizing these effects at the molecular level we showed that mTORC1 inhibition affects S6K1/S6RP but not 4E-BP1 phosphorylation status. In contrast, PI3K/mTOR inhibitors (NVP-BEZ235, LY294002) in addition to S6K1 also inhibit phosphorylation of 4E-BP1. Rapamycin insensitive regulation of 4E-BP1 has been described only recently by other groups e.g. in glioma cells, myoblasts and acute myeloid leukemia when comparing effects of rapamycin and NVP-BEZ235 treatment [45], [46], [47]. An explanation for this observation might be the incomplete inactivation of mTORC1 by rapamycin or its derivatives such as RAD001 or CCI-779 [48]. Here, we suggest an alternative explanation in which 4E-BP1 phosphorylation is regulated directly or indirectly by PI3K but not via Akt-mTORC1 (Figure 7). Evidence for this proposed model resulted from the observation that two different inhibitors of PI3K/mTOR regulate the mTORC1 downstream target S6K1 at different concentrations than Akt/4E-BP1 phosphorylation (Figure 1B,C, 3A). Different kinetics in inactivating mTOR or PI3K has been described for these compounds but the functional consequence on S6K1 and 4E-BP1 has not been described to date. In addition to chemical compounds, our data show that silencing of mTOR or Akt expression both resulted in suppression of S6K1 but not 4E-BP1 phosphorylation (Figure 3B,C). It has been demonstrated that an incomplete knockdown of mTOR still permits phosphorylation of mTOR substrates [49]. In our experiments, knockdown rates were >90% but more important we observed dephosphorylation of S6K1, indicating a successful functional suppression of Akt and mTOR in our experiments. Further studies have to reveal the molecular mechanism that transduce signaling from PI3K to 4E-BP1 and if this observation is a general mechanism in urothelial carcinoma or limited to the specific genetic background of the cell lines examined.

In tumor growth, 4E-BP1 is suggested to integrate signals from the PI3K and the MAPK signaling pathway and might be a potential target for therapy [15]. Our results support this view of 4E-BP1. According to our data S6K1 and 4E-BP1 proteins are to a similar extent involved in regulating cell proliferation and the combined inhibition of both is crucial for effective inhibition of cellular growth (Figure 6B) [15], [50]. We conclude that the superior effect of NVP-BEZ235 on cell proliferation might be explained by the regulation of both S6K1 and 4E-BP1 since one protein is downstream of Akt/mTOR and the other downstream of PI3K. This finding has important implications for the design of anti-tumor therapies in UC. Clearly, it raises the question whether rapamycin analogues are as appropriate for therapy in this tumor as in other entities. The limited clinical efficacy of RAD001 apparent from the preliminary results of a phase II study of patients with platinum-refractory metastatic UC is in keeping with these conclusions [51].

Akt has been described as a key molecule for the regulation of numerous processes including cell cycle progression, survival and apoptosis and is therefore considered a hopeful candidate for targeted therapies [13]. Results from clinical trials with Akt blocking small molecule inhibitors were not as successful as expected indicating an urgent requirement for a better understanding of Akt biology [13], [52]. We confirmed that RAD001 treatment resulted in an S6K1-dependent feedback loop that hyperphosphorylated Akt (Figure 2) [17]. Our results support also a two step model in which mTORC2 has an important role by first phosphorylating S473 which then facilitates phosphorylation of T308 (Figure 2D) [10], [49], [53]. Interestingly, treatment of cells with NVP-BEZ235 inhibited phosphorylation of S6K1 and 4E-BP1 effectively over time whereas the initial dephosphorylation of Akt reversed at prolonged treatment, a phenomenon that has been also observed by other groups, e.g. in human gliomas [24], [54] (Figure 2C). The sustained dephosphorylation of S6K1 could be explained by the different activity of NVP-BEZ235 towards mTOR. However, the re-activation of Akt would suggest activation of PI3K which in our model could reactivate 4E-BP1 also. Here, further studies are necessary to reveal the precise molecular network that regulates 4E-BP1 phosphorylation status.

An important aspect of Akt activity in UC is its function on cell proliferation. As described, phosphorylated Akt induces a positive impulse towards cell proliferation but we observe the opposite (Figure 4) [13]. The strategies we used to interfere with the PI3K pathway blocked Akt downstream elements such as mTOR or S6K1. However, the regulation of apoptosis represents a different branch in Akt signaling and thus phosphorylated Akt probably prevented activation of caspases, which would explain our results (Figure 5C). Clearly, further studies of the circuitry of PI3K, Akt, mTOR and their downstream targets in UC are necessary for defining the best therapeutic target molecules and also stratification marker for therapy in this pathway.

Crosstalk between the MAPK and PI3K signaling pathways has been established in recent years and we could also confirm that either pathway can partially substitute for the failure of the other in UC cell lines [14]. In the current model, Ras regulates activation of PI3K whereas ERK1/2 can trigger mTORC1 activity via TSC2 inhibition or direct interaction with its raptor subunit [55]. Inhibition or genetic deletion of mTOR or S6K1 in mouse embryonic fibroblasts or breast cancer, but also in murine diabetes models resulted in Ras-MAPK activation via insulin receptor substrate-1 (IRS) [17], [18]. In UC, silencing of Ras expression by siRNAs resulted also in reduced phosphorylation of Akt [32]. In our study, inactivation of mTORC1-S6K1 resulted in upregulation of MAPK signaling only after extended exposure to RAD001 or silencing of S6K1 expression (Figure 4). In contrast, when in addition to mTOR also PI3K activity was suppressed by using NVP-BEZ235, instant activation of the MAPK signaling pathway was observed (Figure 4A). We speculate that crosstalk from PI3K/Akt/mTOR to the MAPK signaling pathway might involve two distinct mechanisms. In the first scenario inactivation of PI3K directly results in activation of the MAPK pathway. In the second one, when downstream components such as mTORC1/2 and S6K1 are inhibited the delayed upregulation of MAPK activity might constitute instead an indirect response.

When interfering with MAPK activity using U0126, hyperactivation of Akt was observed but unlike postulated in the current model no or only weak responses on mTOR, S6K1 or 4E-BP1 phosphorylation status were evident (Figure 4C). This might signify that dependent on the stimulus Akt activation does not necessary result in mTOR activation but that other factors and molecular checkpoints are involved that are not activated in our experimental approach.

Based on our data, we suggest for urothelial carcinoma a molecular model depicted in Figure 7 for the molecular interplay of both pathways. Accordingly, we suggest that activation status of Akt, S6K1, 4E-BP1 might be a minimum requirement for stratification of patients that could benefit from a therapy that targets these molecular elements in the PI3K signaling pathway. In particular 4E-BP1 might be a worthwhile new target for cancer therapy. The best benefit for metastatic UC patients might be achieved by the parallel inhibition of 4E-BP1 and S6K1 activity probably in combination with MAPK inhibitors. In mouse tumor xenograft models, the combination of NVP-BEZ235 with MAPK inhibitors has been described for several tumor entities with very promising results and no detectable additive toxicities for the animals [56].
